# Synthesis and luminescence properties of Mn-doped Cs_2_KBiCl_6_ double perovskite phosphors

**DOI:** 10.1186/s11671-023-03820-w

**Published:** 2023-03-13

**Authors:** Qinghua Zhong, Shuai Yu, Quanwei Zheng, Leilei Zhang, Xiaowen Zhang

**Affiliations:** 1 School of Electronics and Information Engineering, South China Normal University, Foshan, 528225 People’s Republic of China; 2 School of Materials Science and Engineering & Guangxi Key Laboratory of Information Materials, Guilin University of Electronic Technology, Guilin, 541004 People’s Republic of China

**Keywords:** Double perovskite phosphor, Cs_2_KBiCl_6_, Mn doping, Orange-red emission, Photoluminescence

## Abstract

Novel lead-free double perovskite phosphors of Mn-doped Cs_2_KBiCl_6_ (Cs_2_KBiCl_6_:Mn^2+^) have been facilely synthesized by using typical hydrothermal method. X-ray diffraction, scanning electron microscope, X-ray photoelectron spectroscopy, electron paramagnetic resonance, and photoluminescence measurements confirm that the synthesized Cs_2_KBiCl_6_:Mn^2+^ phosphors behave double perovskite structure, good morphology, excellent stability, and superior optical properties. An optimal doping concentration of Mn/Bi = 0.4 is achieved in Cs_2_KBiCl_6_:Mn^2+^ phosphors, showing maximum photoluminescence quantum yield of 87.2%, lifetime of 0.98 ms, and orange-red fluorescence with the emission peak at 595 nm under UV light excitation. The probable luminescence mechanism could be ascribed to excitation energy transferring from Cs_2_KBiCl_6_ to Mn, and accordingly contributing to the ^4^T_1_–^6^A_1_ transition of the Mn d electron. Superb optical properties provide much room for in-depth fluorescence researches and potential applications of Cs_2_KBiCl_6_:Mn^2+^ phosphors.

**Supplementary Information:**

The online version contains supplementary material available at 10.1186/s11671-023-03820-w.

## Introduction

Newly emerged double perovskite phosphors are highly spotlighted due to their potential applications to stylish displays and lighting. The lead-free metal halide double perovskite of A_2_M^I^M^III^X_6_ (A = Cs^+^; M^I^ = Cu^+^, Ag^+^, Na^+^, K^+^; M^III^ = Bi^3+^, Sb^3+^, In^3+^; X = Cl, Br, I) possesses specific 3D electronic structure, good stability, and environment-friendly sustainability, receiving increasing attentions in luminescence researches [1–3]. Cs_2_AgBiX_6_ (X = Cl, Br) is the first reported fluorescence material with double perovskite structure in 2016 [4–6]. Sb- or Mn-doped Cs_2_NaInCl_6_ are also good representatives of double perovskite phosphors [3, 7–9]. Cs_2_KBiCl_6_ shows similar double perovskite structure and could be considered as a promising phosphor. At present, to the best of our knowledge, there are rare reports on Cs_2_KBiCl_6_ and its composites. However, it is predictably deduced that pristine Cs_2_KBiCl_6_ just like Cs_2_NaInCl_6_ usually produces negligible or undetectable fluorescence under UV light excitation due to the parity-forbidden nature [3, 8, 9].

It is well established that the Sb^3+^ or Mn^2+^ doping could break such an intractable parity-forbidden transition mechanism, and thus considerably improving the optical performance. For instance, Cs_2_NaInCl_6_:Sb^3+^ [7–9], Cs_2_NaInCl_6_:Mn^2+^ [3], Cs_2_NaBiCl_6_:Mn^2+^ [10, 11], Cs_2_AgBiX_6_:Mn^2+^ (X = Cl, Br) [12], Cs_2_AgInCl_6_:Mn^2+^ [13], A_2_BAlF_6_:Mn^4+^ (A = Rb, Cs; B = K, Rb) [14] and Cs_2_Na_1−*x*_Ag_*x*_BiCl_6_:Mn^2+^ [15] are well-known double perovskite phosphors. In this study, we explore a novel double perovskite phosphor of Cs_2_KBiCl_6_ with Mn doping (Cs_2_KBiCl_6_:Mn^2+^). Efficient excitation energy transferring from the host matrix of Cs_2_KBiCl_6_ to the dopant of Mn is occurred, contributing to the ^4^T_1_–^6^A_1_ transition of the Mn d electron, and yielding orange-red fluorescence with the emission peak at 595 nm and maximum photoluminescence quantum yield (PLQY) of 87.2%. Superb optical properties provide much room for in-depth fluorescence researches and potential applications of Cs_2_KBiCl_6_:Mn^2+^ phosphors.

## Experimental details

Chemical materials of CsCl (99.99%, Alfa Aesar), KCl (99.99%, Alfa Aesar), BiCl_3_ (99.99%, Alfa Aesar), MnCl_2_·4H_2_O (99%), HCl (37%), and (CH_3_)_2_CHOH (99.9%) were commercially purchased and used as raw materials without further purification. Firstly, CsCl, BiCl_3_, and MnCl_2_·4H_2_O were directly dissolved into HCl solution, preparing 1 mol/L CsCl solution, 0.5 mol/L BiCl_3_ solution, and 1 mol/L MnCl_2_ solution, respectively. Then, Cs_2_KBiCl_6_:Mn^2+^ powders were synthesized by using a typical hydrothermal method. 0.03727 g KCl powders, 1 mL CsCl solution, 1 mL BiCl_3_ solution, and *x* mL MnCl_2_ solution (*x* = 0, 0.1, 0.2, 0.3, 0.4, 0.5, corresponding to Mn/Bi = 0, 0.2, 0.4, 0.6, 0.8, 1, respectively, in the final products of Cs_2_KBiCl_6_:Mn^2+^) were sequentially added into a stainless-steel autoclave equipped with a Teflon liner. Adding extra HCl solution till the total volume reaches 5 mL. After reaction at 180 °C for 2 h, the precipitates were washed with (CH_3_)_2_CHOH and dried at 60 °C for 6 h. The final products of Cs_2_KBiCl_6_:Mn^2+^ powders were obtained.

X-ray diffraction (XRD, Bruker D8 Advance), X-ray photoelectron spectroscopy (XPS, Thermo Escalab 250Xi), and scanning electron microscope (SEM, JEOL JEM-2100F) equipped with energy-dispersive X-ray spectroscopy (EDS) were used for analyzing the crystal phase structure, element distribution mapping, chemical state, and surface morphology. Brooke 300 200 electron paramagnetic resonance (EPR) spectrometer with an X-band microwave frequency of 10 GHz was used for investigating the EPR characteristics. PerkinElmer Lambda 365 UV–vis Spectrophotometer, and Edinburgh Instruments FS5 spectrofluorometer equipped with an integrating sphere were used for measuring UV–vis absorption spectra, PL spectra, PLQYs, and time-resolved PL (TRPL) decay profiles. The PL spectra were measured under 365 nm excitation, while the TRPL profiles were obtained using excitation wavelength of 430 nm and emission wavelength of 590 nm.

## Results and discussion

**Fig. 1:**
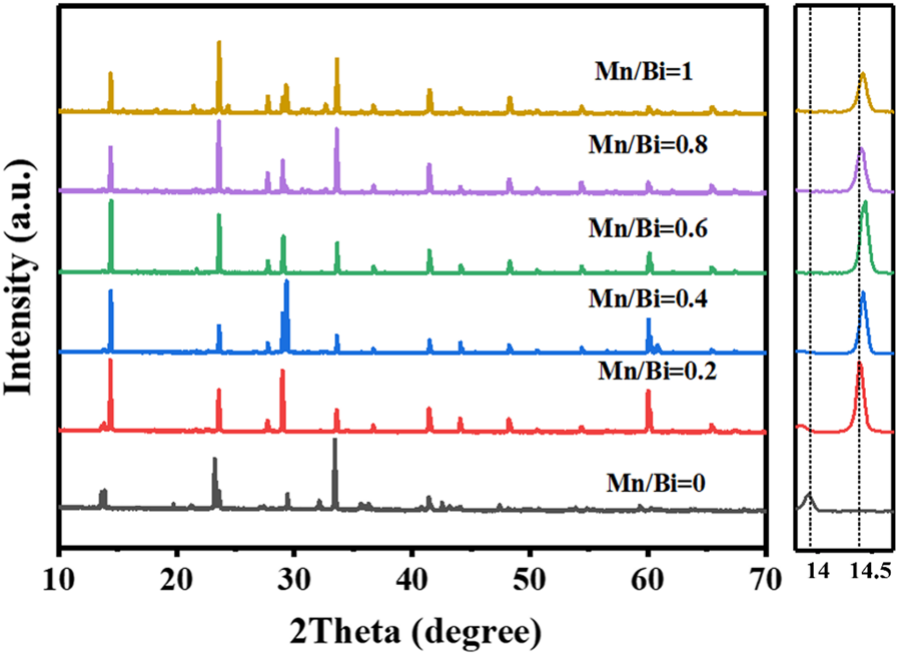
XRD patterns of Cs_2_KBiCl_6_:Mn^2+^ (Mn/Bi = 0, 0.2, 0.4, 0.6, 0.8 and 1) powders

Figure 1 shows the XRD patterns of Cs_2_KBiCl_6_:Mn^2+^ (Mn/Bi = 0, 0.2, 0.4, 0.6, 0.8 and 1). It is observed that the Cs_2_KBiCl_6_:Mn^2+^ remains similar crystal structure as Cs_2_KBiCl_6_, although the diffraction peaks around 2θ = 14° slightly shift to high angles after doping Mn. The ionic radius of Mn^2+^ (0.67 Å) is smaller than those of K^+^ (1.38 Å) and Bi^3+^ (1.03 Å) [3, 16–18]. The Mn^2+^ substitution results in lattice shrinkage, which accounts for the high-angle shifting of diffraction peaks. The radius of Mn^2+^ is largely deviated from Cs^+^ (1.67 Å) [18], it is boldly speculated that the Mn^2+^ occupies Bi^3+^ site or K^+^ site and forms a double perovskite architecture. The diffraction peak at 2*θ* = 14.4° shifts to higher angle in Mn/Bi = 0.4 sample as compared with the Mn/Bi = 0.2 sample. It indicates that more Mn^2+^ substitution results in more excitation energy transferring from Cs_2_KBiCl_6_ matrix to the doped Mn^2+^ and enhances PLQYs. There is no observable high-angle shifting with further increasing Mn^2+^ concentrations (Mn/Bi = 0.6, 0.8 and 1), suggesting that excessive Mn^2+^ doping attenuates PL intensity as a result of concentration-induced quenching effect.

The SEM images of Cs_2_KBiCl_6_ and Cs_2_KBiCl_6_:Mn^2+^ (Mn/Bi = 0.4) are shown in Fig. S1 in Supporting Information. It is observed that both show micron size and similar appearance with certain flake shape. More flaky Cs_2_KBiCl_6_:Mn^2+^ (Mn/Bi = 0.4) maybe caused by the hygroscopicity and deliquesce of MnCl_2_·4H_2_O. The EDS mappings (Fig. S2 in Supporting Information) show the elements distribution of Cs, K, Bi, Cl, and Mn of Cs_2_KBiCl_6_:Mn^2+^ (Mn/Bi = 0.4). It is obvious that the components are uniformly distributed and the Mn^2+^ is fully doped into the host matrix of Cs_2_KBiCl_6_.

The XPS core level spectra of Cs_2_KBiCl_6_ and Cs_2_KBiCl_6_:Mn^2+^ (Mn/Bi = 0.4) are shown in Fig. S3 in Supporting Information. The element signals of Cs, K, Bi, Cl and Mn are distinctly detected. The binding energies of Mn 2p are positioned at 641.4 eV and 654.3 eV, corresponding to Mn 2p_3/2_ and Mn 2p_1/2_, respectively. As compared with pristine Cs_2_KBiCl_6_, the signals of K 2p species in Cs_2_KBiCl_6_:Mn^2+^ (Mn/Bi = 0.4) are considerably weakened, may arising from the substitution of Mn^2+^ and/or the alteration of surface states such as carbon absorbing.

**Fig. 2:**
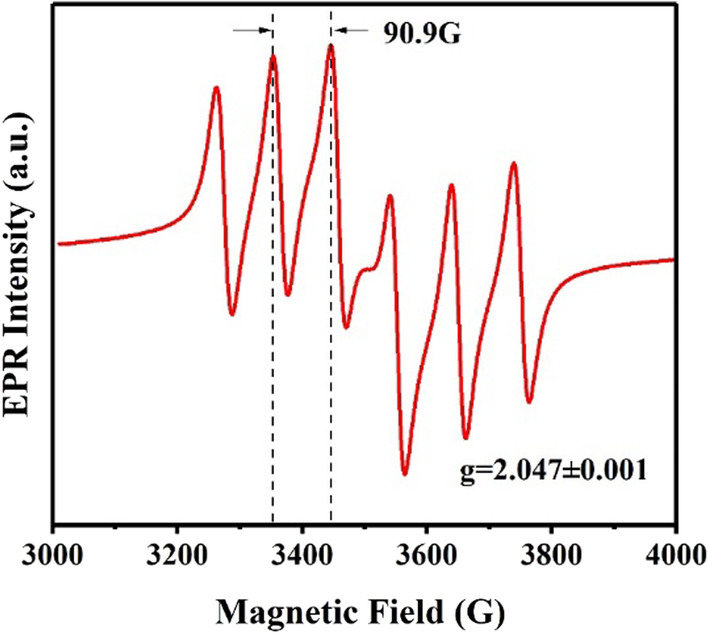
EPR spectrum of Cs_2_KBiCl_6_:Mn.^2+^ (Mn/Bi = 0.4)

The EPR spectrum of Cs_2_KBiCl_6_:Mn^2+^ (Mn/Bi = 0.4) is explored and shown in Fig. 2. A sixfold hyperfine coupling pattern is observed, showing a hyperfine constant A of 90.9 G and a characteristic g-factor of 2.047 ± 0.001. Such specific characteristics are derived from the isotropic hyperfine coupling of Mn^2+^ electron spin state and nuclear spin [19]. The EPR spectrum also suggests that Mn^2+^ ions have been successfully doped into Cs_2_KBiCl_6_ matrix.

The UV–vis absorption spectra of Cs_2_KBiCl_6_:Mn^2+^ (Mn/Bi = 0, 0.2, 0.4, 0.6, 0.8 and 1) are depicted in Fig. S4 in Supporting Information. The pristine Cs_2_KBiCl_6_ shows an absorption peak at about 390 nm (Fig. S4a), owing to the intrinsic self-trapped exciton (STE) absorbance of double perovskite materials. There is no visible emission under UV excitation for pristine Cs_2_KBiCl_6_. With Mn^2+^ doping, some absorption peaks in the visible region (particularly 400–600 nm) are observed (Figs. S4b–f). These newly appeared absorption peaks are originated from the ^4^T_1_–^6^A_1_ transition absorption of Mn^2+^. It indicates that strong lattice vibration reduces the STE emission of Cs_2_KBiCl_6_, and accordingly transferring the excitation energies to Mn^2+^ as well as producing robust (stronger than STE) d–d transition emission of Mn^2+^. In addition, the visible emission peaks position of Cs_2_KBiCl_6_:Mn^2+^ (Mn/Bi = 0.2, 0.4, 0.6, 0.8 and 1) does not change with the variation of Mn concentration (Figs. S4b–f), which also reflects that the fluorescence shows invariant emission peak for Cs_2_KBiCl_6_:Mn^2+^ phosphors.

**Fig. 3:**
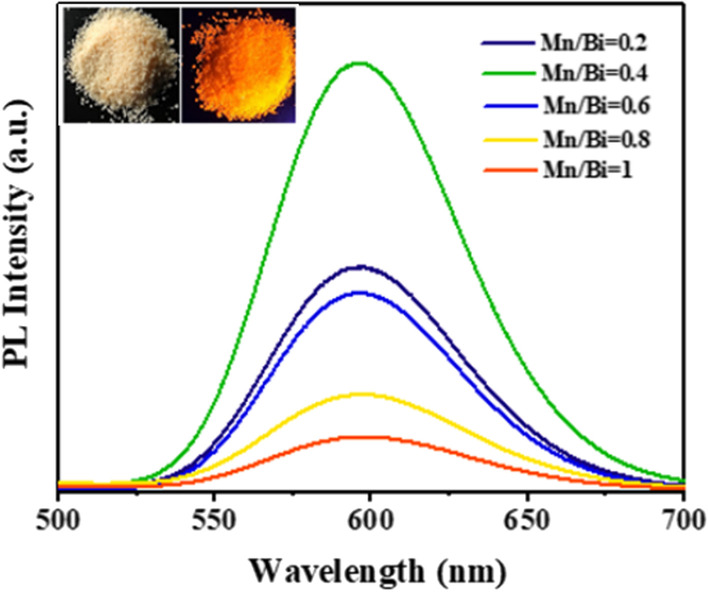
PL intensity of Cs_2_KBiCl_6_:Mn^2+^ (Mn/Bi = 0.2, 0.4, 0.6, 0.8 and 1). Inset shows the images of pristine Cs_2_KBiCl_6_ (left) under visible irradiation and Cs_2_KBiCl_6_:Mn^2+^ (Mn/Bi = 0.4, right) under 365 nm excitation

The fluorescence characteristics of Cs_2_KBiCl_6_:Mn^2+^ phosphors are shown in Fig. 3. Considerable PL with emission peak at 595 nm is observed under 365 nm excitation. Moreover, the PL intensity is gradually increased with increasing Mn concentration and reaches maximum when Mn/Bi = 0.4 in Cs_2_KBiCl_6_:Mn^2+^. Further increasing Mn concentration (Mn/Bi = 0.6, 0.8 and 1) attenuates PL intensity. Excessive Mn doping would increase non-radiative center and cause concentration-induced quenching. The measured PLQYs of Cs_2_KBiCl_6_:Mn^2+^ (Mn/Bi = 0.2, 0.4, 0.6, 0.8 and 1) are shown in Fig. S5 in Supporting Information. The sample with Mn/Bi = 0.4 behaves the highest value of 87.2%, indicating superb optical properties and providing much room for fluorescence researches and potential applications. The insets in Fig. 3 show the Cs_2_KBiCl_6_ image under visible irradiation, since there is no observable fluorescence under UV light excitation. While the Cs_2_KBiCl_6_:Mn^2+^ (Mn/Bi = 0.4) image appears distinct orange-red under 365 nm excitation. The probable emission mechanism in Cs_2_KBiCl_6_:Mn^2+^ could be ascribed to the excitation energy transferring from the Cs_2_KBiCl_6_ matrix to the dopant of Mn^2+^, which is similar to the previously reported Mn-doped double perovskite phosphors [3, 11, 19]. The ^4^T_1_–^6^A_1_ transition from Mn d electron contributes to the orange-red emission of Cs_2_KBiCl_6_:Mn^2+^ under UV light excitation.

**Fig. 4:**
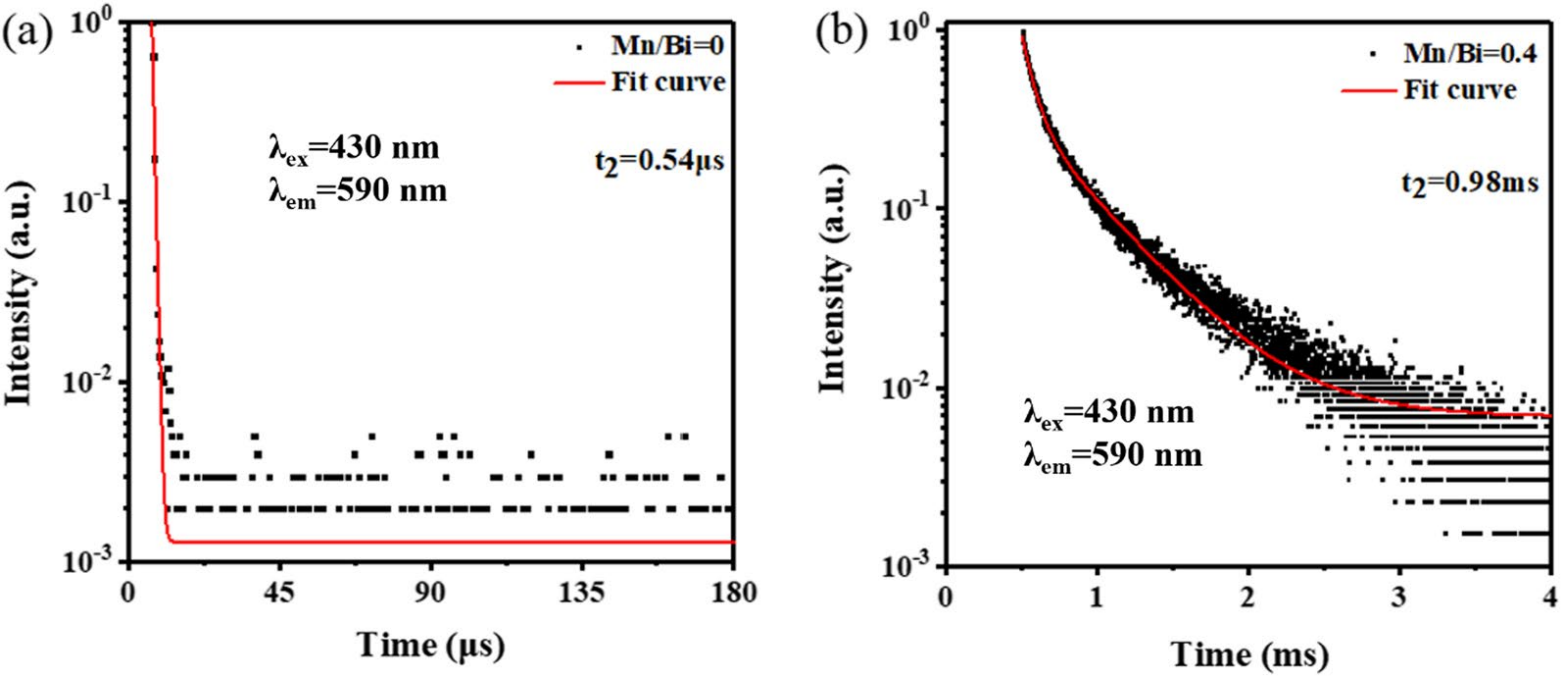
TRPL decay curves of **a** Cs_2_KBiCl_6_ and **b** Cs_2_KBiCl_6_:Mn.^2+^ (Mn/Bi = 0.4)

Figure 4 shows the TRPL decay profiles of Cs_2_KBiCl_6_ and Cs_2_KBiCl_6_:Mn^2+^ (Mn/Bi = 0.4). The lifetime (*τ*_av_) are calculated from the fitted curves using double-exponential function of Eq. (1).

1 $$\tau_{{{\text{av}}}} = {{\mathop \sum \limits_{i} A_{i} \tau_{i}^{2} } \mathord{\left/ {\vphantom {{\mathop \sum \limits_{i} A_{i} \tau_{i}^{2} } {\mathop \sum \limits_{i} A_{i} \tau_{i} }}} \right. \kern-0pt} {\mathop \sum \limits_{i} A_{i} \tau_{i} }}$$

Here *A*_*i*_ and *τ*_*i*_ are the weight coefficient and radiation time, respectively. The fitted results indicate that pristine Cs_2_KBiCl_6_ shows short lifetime of only 0.54 μs, while Cs_2_KBiCl_6_:Mn^2+^ behaves typical millisecond (ms) or sub-ms lifetime of 0.98 ms. The ms lifetime also suggests low concentration of trap states on surface or inside of the Cs_2_KBiCl_6_:Mn^2+^, contributing to larger carrier diffusion length and higher PL intensity. Moreover, it is rationally deduced that the STE emission could be nearly excluded in Cs_2_KBiCl_6_:Mn^2+^ phosphors, and efficient energy transferring from Cs_2_KBiCl_6_ to Mn^2+^ is occurred, since the lifetime of STE emission is usually within the μs range.

The stability of Cs_2_KBiCl_6_:Mn^2+^ (Mn/Bi = 0.4) is investigated via investigating the XRD patterns of fresh Cs_2_KBiCl_6_:Mn^2+^ (Mn/Bi = 0.4) and placing for 6 months in air conditions (Fig. S6 in Supporting Information). The XRD patterns do not show observable difference, suggesting that the crystal phase structure is not changed. It reflects good stability and advances potential applications of Cs_2_KBiCl_6_:Mn^2+^ phosphors.

## Conclusions

Cs_2_KBiCl_6_:Mn^2+^ phosphors have been facilely synthesized by using a typical hydrothermal method. XRD, SEM, XPS and EPR measurements are taken. The experimental results show that synthesized Cs_2_KBiCl_6_:Mn^2+^ phosphors behave double perovskite structure, good morphology, and superior stability. The Mn is effectively doped into the Cs_2_KBiCl_6_ matrix, while the optimal doping concentration is Mn/Bi = 0.4. The PL measurements reveal that Cs_2_KBiCl_6_:Mn^2+^ (Mn/Bi = 0.4) shows maximum PLQY of 87.2%, lifetime of 0.98 ms, and distinct orange-red fluorescence with the emission peak at 595 nm under 365 nm excitation, owing to excitation energy transferring from Cs_2_KBiCl_6_ to Mn, and accordingly contributing to the ^4^T_1_–^6^A_1_ transition of the Mn d electron.

## Supplementary Information

Additional file1 (DOCX 1701 KB)

## Data Availability

The data that support the findings of this study are available from the corresponding author upon reasonable request.

## References

[1] Chu L, Ahmad W, Liu W, Yang J, Zhang R, Sun Y, Yang JP, Li XA (2019). Lead free halide double perovskite materials: a new superstar toward green and stable optoelectronic applications. Nano-Micro Lett.

[2] Luo JJ, Hu MC, Niu GD, Tang J (2019). Lead-free halide perovskites and perovskite variants as phosphors toward light-emitting applications. ACS Appl Mater Interfaces.

[3] Zhang L, Xie X, Li D, Yuan Y, Xue X, Li Q, Xu J, Wang H, Hu F, Zhang X (2021). Investigation on lead-free Mn-doped Cs_2_NaInCl_6_ double perovskite phosphors and their optical properties. Opt Mater.

[4] McClure ET, Ball MR, Windl W, Woodward PM (2016). Cs_2_AgBiX_6_ (X=Br, Cl)-New visible light absorbing, lead-free halide perovskite semiconductors. Chem Mater.

[5] Volonakis G, Filip MR, Haghighirad AA, Sakai N, Wenger B, Snaith HJ, Giustino F (2016). Lead-free halide double perovskites via heterovalent substitution of noble metals. J Phys Chem Lett.

[6] Slavney AH, Hu T, Lindenberg AM, Karunadasa HI (2016). A bismuth-halide double perovskite with long carrier recombination lifetime for photovoltaic application. J Am Chem Soc.

[7] Gray MB, Hariyani S, Strom TA, Majher JD, Brgoch J, Woodward PM (2020). High efficiency blue photoluminescence in the Cs_2_NaInCl_6_:Sb^3+^ double perovskite phosphor. J Mater Chem C.

[8] Zeng RS, Zhang LL, Xue Y, Ke B, Zhao Z, Huang D, Wei QL, Zhou WC, Zou BS (2020). Highly efficient blue emission from self-trapped excitons in stable Sb^3+^- doped Cs_2_NaInCl_6_ double perovskites. J Phys Chem Lett.

[9] Noculak MA, Morad V, McCall KM, Yakunin S, Shynkarenko Y, Wörle M, Kovalenko MV (2020). Bright blue and green luminescence of Sb(III) in double perovskite Cs_2_MInCl_6_ (M=Na, K) matrices. Chem Mater.

[10] Yao MM, Wang L, Yao JS, Wang KH, Chen C, Zhu BS, Yang JN, Wang JJ, Xu WP, Zhang Q, Yao HB (2020). Improving lead-free double perovskite Cs_2_NaBiCl_6_ nanocrystal optical properties via ion doping. Adv Optical Mater.

[11] Majher JD, Gray MB, Strom TA, Woodward PM (2019). Cs_2_NaBiCl_6_:Mn^2+^—a new orange-red halide double perovskite phosphor. Chem Mater.

[12] Chen N, Cai T, Li WH, Hills-Kimball K, Yang HJ, Que MD, Nagaoka Y, Liu ZY, Yang D, Dong AG, Xu CY, Zia R, Chen O (2019). Yb- and Mn-doped lead-free double-perovskite Cs_2_AgBiX_6_ (X=Cl^−^, Br^−^) nanocrystals. ACS Appl Mater Interfaces.

[13] Liu Y, Nag A, Manna L, Xia Z (2021). Lead-free double perovskite Cs_2_AgInCl_6_. Angew Chem Int Ed.

[14] Deng TT, Song EH, Zhou YY, Wang LY, Zhang QY (2017). Tailoring photoluminescence stability in double perovskite red phosphors A_2_BAlF_6_:Mn^4+^ (A=Rb, Cs; B=K, Rb) via neighboring-cation modulation. J Mater Chem C.

[15] Ke B, Zeng RS, Zhao Z, Wei QL, Xue XG, Bai K, Cai CX, Zhou WC, Xia ZG, Zou BS (2020). Homo/Hetero-Valent doping mediated self-trapped excitons emission and energy transfer in Mn-doped Cs_2_Na_1__−__x_Ag_x_BiCl_6_ double perovskites. J Phys Chem Lett.

[16] Shannon RD (1976). Revised effective ionic radii and systematic studies of interatomic distances in halides and chalcogenides. Acta Crystallogr A.

[17] Zhou J, Rong X, Zhang P, Molokeev MS, Wei P, Liu Q, Zhang X, Xia Z (2019). Manipulation of Bi^3+^/In^3+^ transmutation and Mn^2+^-doping effect on the structure and optical properties of double perovskite Cs_2_NaBi_1-x_In_x_Cl_6_. Adv Opt Mater.

[18] Li C, Wang A, Xie L, Deng X, Liao K, Yang J, Li T, Hao F (2019). Emerging alkali metal ion (Li^+^, Na^+^, K^+^ and Rb^+^) doped perovskite films for efficient solar cells: recent advances and prospects. J Mater Chem A.

[19] Liu XY, Xu X, Li B, Yang LL, Li Q, Jiang H, Xu DS (2020). Tunable dual-emission in monodispersed Sb^3+^/Mn^2+^ codoped Cs_2_NaInCl_6_ perovskite nanocrystals through an energy transfer process. Small.

