# Supplementary material for: Synthesis and luminescence properties of Mn-doped Cs2KBiCl6 double perovskite phosphors
Source: Discov Nano. 2023 Mar 13;18(1):42. doi: 10.1186/s11671-023-03820-w (PMC10214904; doi:10.1186/s11671-023-03820-w)
Supplement: Supplementary file 1 — Additional file1 (DOCX 1701 KB) [file 11671_2023_3820_MOESM1_ESM.docx]

**Supporting Information**


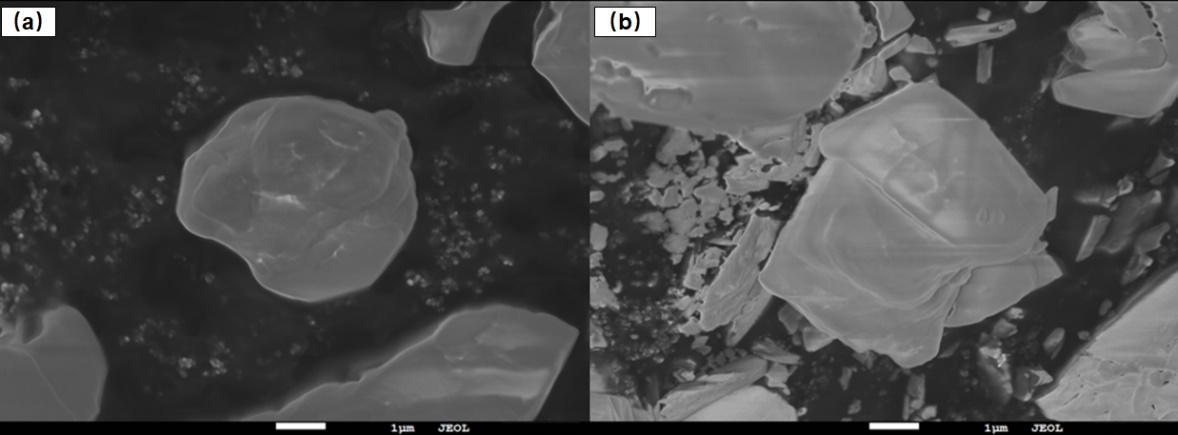


**Fig. S1** SEM images of (a) Cs_2_KBiCl_6_ and (b) Cs_2_KBiCl_6_:Mn^2+^ (Mn/Bi=0.4).


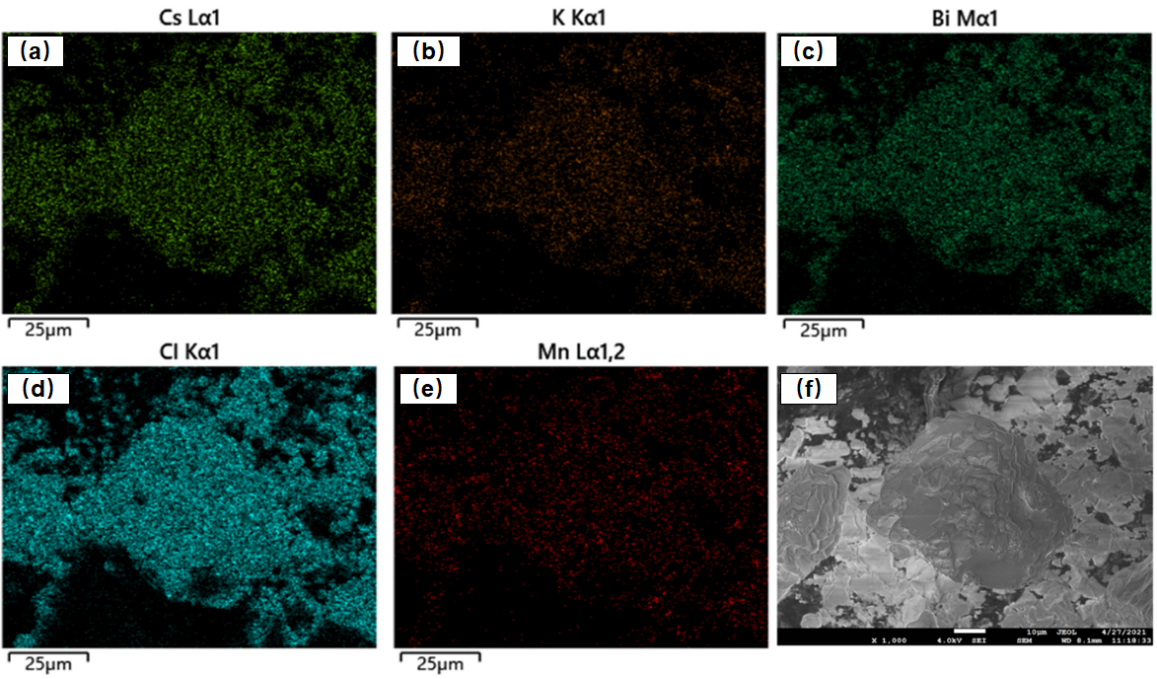


**Fig. S2** Elements distribution mappings of (a) Cs, (b) K, (c) Bi, (d) Cl, (e) Mn from EDS measurements. (f) The corresponding SEM morphology of Cs_2_KBiCl_6_:Mn^2+^ (Mn/Bi=0.4).


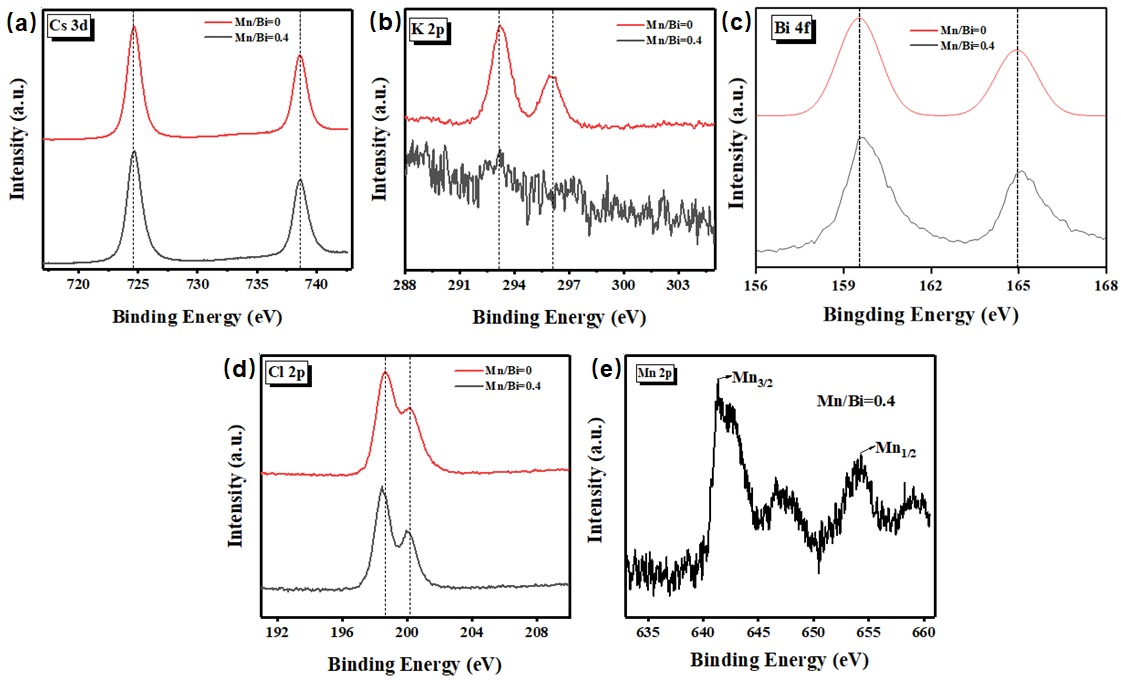


**Fig. S3** XPS core level spectra of (a) Cs 3d, (b) K 2p, (c) Bi 4f, and (d) Cl 2p for Cs_2_KBiCl_6_:Mn^2+^ (Mn/Bi=0 and 0.4). (e) The Mn 2p spectra of Cs_2_KBiCl_6_:Mn^2+^ (Mn/Bi=0.4).


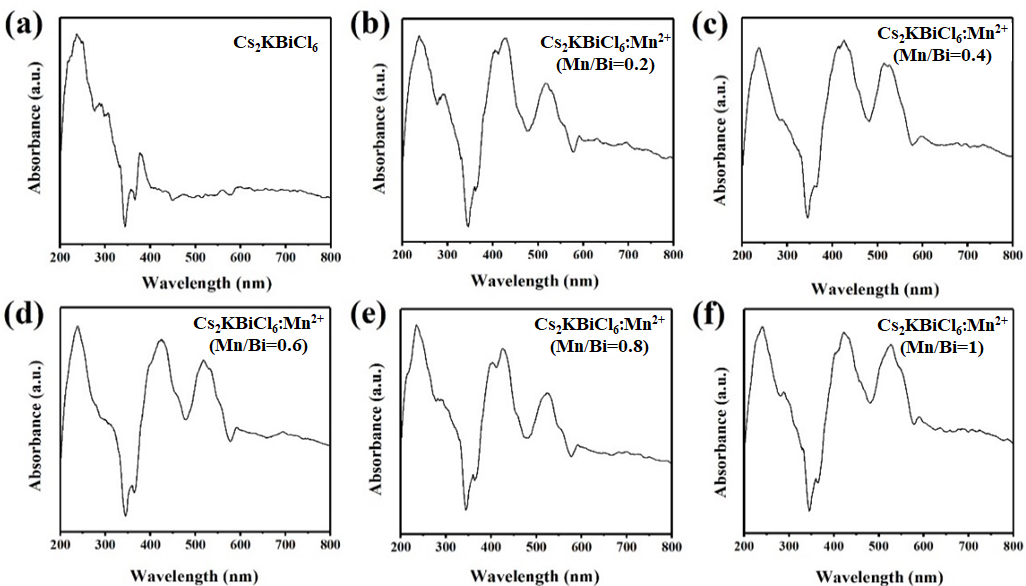


**Fig. S4** UV-vis absorption spectra of Cs_2_KBiCl_6_:Mn^2+^ (Mn/Bi=0, 0.2, 0.4, 0.6, 0.8 and 1).


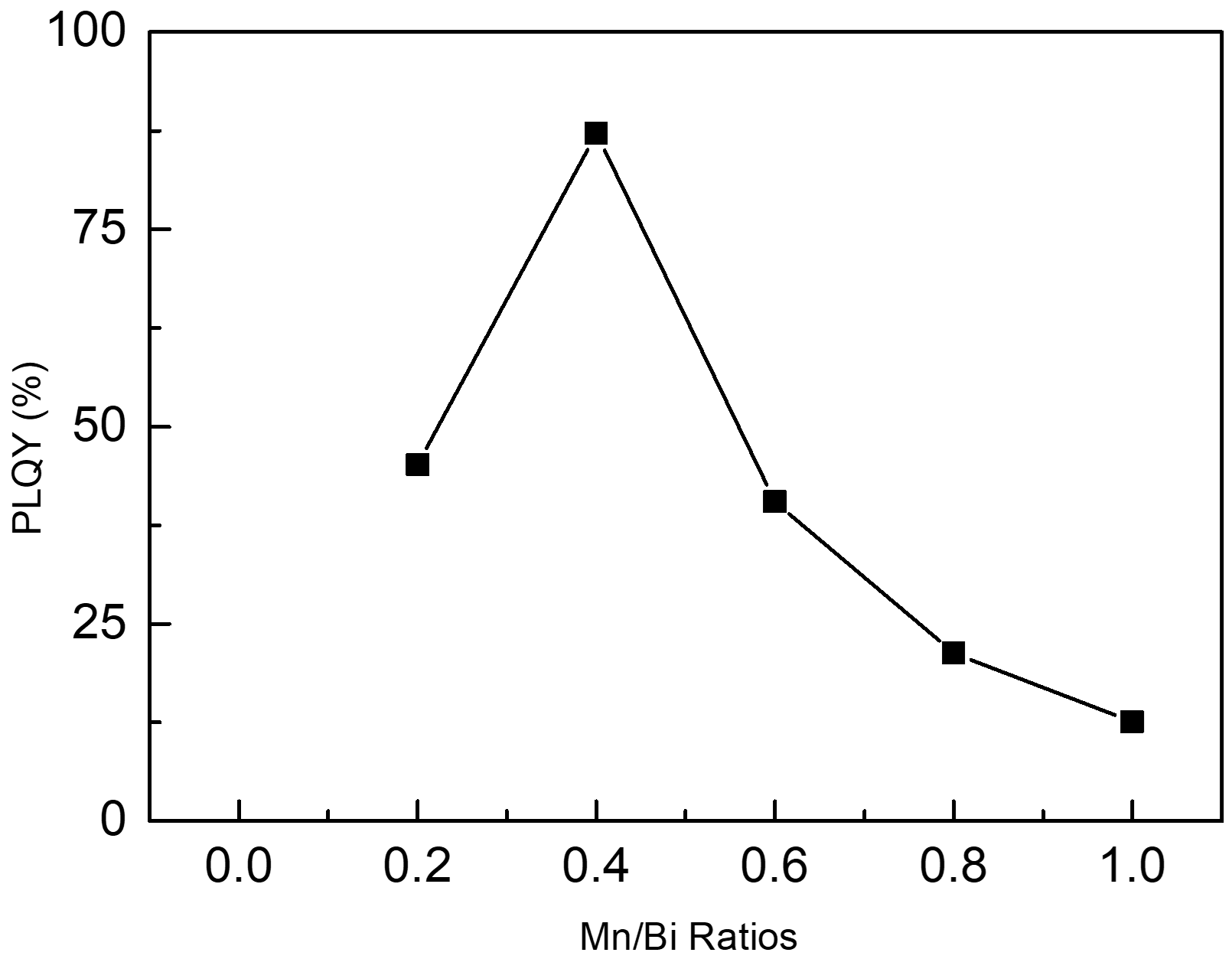


**Fig. S5** Measured PLQYs of Cs_2_KBiCl_6_:Mn^2+^ (Mn/Bi=0.2, 0.4, 0.6, 0.8 and 1).


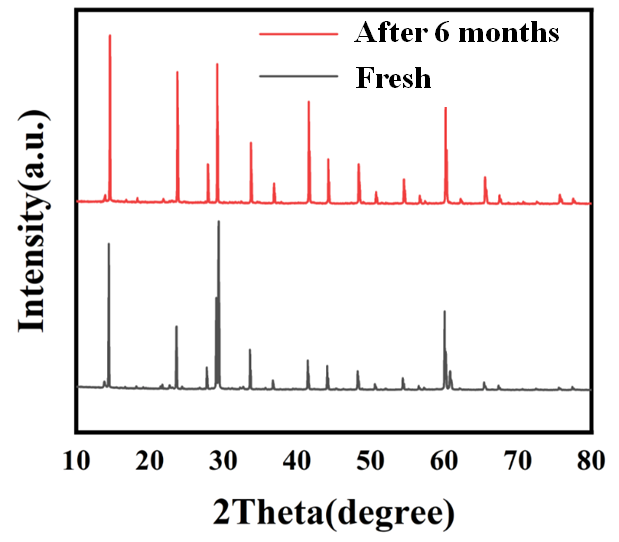


**Fig. S6** XRD patterns comparison of fresh Cs_2_KBiCl_6_:Mn^2+^and placing for 6 months in air conditions.
